# Physiological role of bicarbonate in microbes: A double-edged sword?

**DOI:** 10.1080/21505594.2025.2474865

**Published:** 2025-03-06

**Authors:** Ashok Aspatwar, Jenny Parkkinen, Seppo Parkkila

**Affiliations:** a Faculty of Medicine and Health Technology, Tampere University, Tampere, Finland; b Department of Clinical Chemistry, Fimlab Laboratories PLC, Tampere University Hospital, Tampere, Finland

**Keywords:** Bicarbonate, virulence, antibacterial activity, antibiotics, biofilm, bacteria

## Abstract

HCO_3_^–^ is involved in pH homoeostasis and plays a multifaceted role in human health. HCO_3_^–^ has been recognized for its antimicrobial properties and is pivotal in bacterial antibiotic susceptibility. Notably, the interconversion between CO_2_ and HCO_3_^–^, facilitated by the enzyme carbonic anhydrase (CA), is crucial in tissues infected by pathogens. Studies have highlighted the antimicrobial potency of CA inhibitors, emphasizing the importance of this enzyme in this area. The potential of HCO_3_^–^ as an antibiotic adjuvant is evident; its ability to increase virulence in pathogens such as *Enterococcus faecalis* and *Mycobacterium tuberculosis* requires meticulous scrutiny. HCO_3_^–^ modulates bacterial behaviours in diverse manners: it promotes *Escherichia coli* O157:H7 colonization in the human gut by altering specific gene expression and, with *Pseudomonas aeruginosa*, amplifies the effect of tobramycin on planktonic cells while promoting biofilm formation. These multifaceted effects necessitate profound mechanistic exploration before HCO_3_^–^ can be considered a promising clinical adjuvant.

## GRAPHICAL ABSTRACT

**Figure:**
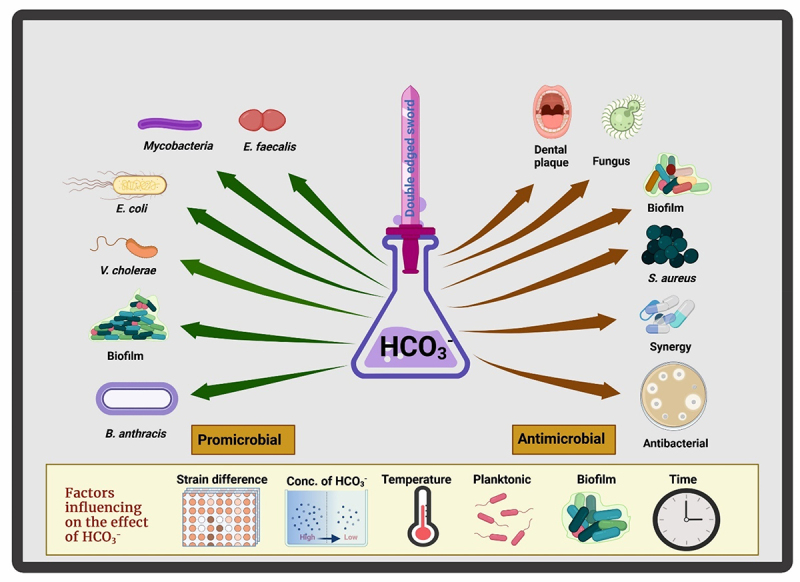

## Introduction

HCO_3_^–^ plays a multifaceted role in both human health and disease. Serving as the primary pH buffer in mammals, it maintains the acid-base balance, which is essential for normal cellular function [1]. The stability of the body’s pH is critical, and even minor deviations can lead to severe metabolic dysfunction [1]. HCO_3_^–^ production is a fascinating biochemical process. Carbon dioxide (CO₂), a byproduct of various metabolic processes, is present in the body and combines with water (H₂O). This reaction is catalysed by an enzyme known as carbonic anhydrase (CA), resulting in the formation of HCO₃^−^ and a proton (H^+^). Given the importance of this reaction in pH regulation, the widespread use of CA enzymes is unsurprising. Interestingly, bacteria that have evolved various physiological mechanisms in eggs possess multiple CA genes, emphasizing their importance [1]. Thus, the role of HCO_3_^–^ extends beyond human physiology.

The pathogenic bacteria responsible for various infectious diseases have evolved to thrive in HCO_3_^–^-rich environments. This underlines the importance of HCO₃^−^ in understanding host-pathogen interactions [2]. The antibacterial and antifungal properties of HCO₃^−^ have been recognized for years [3]. Historically, research since the 1980s has highlighted its efficacy against periodontal pathogens, leading to its incorporation into dental hygiene products [4]. Over the years, its antimicrobial effects against a plethora of pathogens, such as *E. coli*, *Lactobacillus plantarum (L. plantarum)*, *Staphylococcus aureus* (*S. aureus*), and *P. aeruginosa*, and even fungi, such as *Saccharomyces cerevisiae* (*S. cerevisiae*) and *Hansenula wingei* (*H. wingei*), have been documented [5]. However, the exact molecular mechanisms remain the subject of intensive research.

HCO₃^−^ modulates the pH gradient across bacterial membranes, affecting both gram-positive and gram-negative bacteria. Notably, HCO₃^−^ hinders biofilm formation by *P. aeruginosa*, a notorious pathogen, thereby hampering its virulence [6]. The enhancement of antibiotic potency by HCO₃^−^ is truly remarkable. In addition to enhancing the effects of traditional antibiotics, HCO_3_^–^ increases the efficacy of antimicrobial peptides. Thus, it could be an adjunct to antibiotic treatment and should be considered when new antibacterial drugs are developed [7]. Although HCO_3_^–^ enhances the efficacy of antibiotics, it also promotes the growth of certain pathogenic bacteria. For example, *M. tuberculosis*, the causative agent of tuberculosis, flourishes in macrophage compartments at various pH values. HCO_3_^–^ contributes to the virulence of this pathogen [8]. Moreover, HCO_3_^–^ positively influences the export of extracellular DNA in several non-tuberculous mycobacteria, an effect that is pH-independent [8,9]. Hence, the dual role of HCO₃^−^ as both a supporter and an inhibitor of bacterial growth necessitates a cautious approach when considering its therapeutic applications [9,10].

HCO₃^−^ has a complex role in bacterial interactions, enhancing antibiotic effectiveness and promoting virulence gene expression in various bacteria, including *B. anthracis* and *V. cholerae* [11]. Interestingly, CA inhibitors can inhibit this HCO_3_^–^-induced virulence. Furthermore, although HCO_3_^–^synergizes with tobramycin against *P. aeruginosa* in one context, it also promotes biofilm growth [12]. Given these contradictory findings, a nuanced understanding of the molecular role of HCO_3_^–^ is essential before its use as an antibiotic adjuvant. In conclusion, the roles of HCO_3_^–^ in health, disease, and therapeutic applications are multifaceted. With a surge in HCO_3_^–^-related studies, the future promises more insights into its potential benefits and challenges.

## Physiological role of HCO_3_^–^ in health and diseases in humans

HCO_3_^–^ is produced by the reversible hydration of CO_2_ by CAs (CO_2_ + h_2_O ⇌ HCO_3_^−^ + h^+^); it is one of the major anions in humans, and the main function of HCO_3_^–^ is pH homoeostasis [1,13]. In addition to intracellular fluid, body secretions such as saliva, tears, aqueous humour, pancreatic juice, intestinal fluid, airway surface liquid (ASL), and cerebrospinal fluid contain different concentrations of HCO_3_^−^, which then controls the activity and stability of the proteins dissolved in these fluids [14]. Many epithelia specialize in HCO_3_^–^-rich fluid secretion, which generates flow, alters viscosity, controls pH, and potentially protects luminal and intracellular structures from chemical stress and infections. In hypertensive adults, a higher concentration of HCO_3_^–^ is associated with better cognitive and executive performance than a lower concentration of HCO_3_^–^, suggesting that a low concentration of HCO_3_^–^ is harmful to neuronal activity [15]. HCO_3_^–^ plays an important role in the capacitation of sperm by alkalizing the sperm cytosol, which is needed for plasma membrane hyperpolarization and hyperactivation of motility and acts as a second messenger, triggering sperm hypermobility and the acrosomal reaction [16]. The transport of HCO_3_^–^ through anion channels influences the membrane potential of epithelial cells. In the nervous system, HCO_3_^–^ transport through ion channels, including glycine and GABA receptors, is needed for the regulation of neuronal excitability [14]. In mucosal epithelia, HCO_3_^–^ promotes the solubilization and expansion of mucin molecules [14]. HCO_3_^–^ has been shown to play a role in cardiac function in isolated cardiomyocytes, which show increased contractility [17].

Studies have shown that rhinoviruses and coronaviruses enter host cells via fusion with cellular membranes at low pH [18]. In SARS-CoV-2-infected patients, nasal irrigation and oral rinsing with 59.5 mm HCO_3_^–^ solution cleared the virus [18]. It is believed that HCO_3_^–^ increases the pH in vesicles/endosomes and inhibits the nucleocapsid release of SARS-CoV-2, which requires an acidic endosomal environment for uncoating. In addition, in people with flu, there was a decrease in the levels of HCO_3_^−^ both in blood plasma and in tissues, suggesting that proper regulation of HCO_3_^–^ protects humans from infections.

Defects in either the production or transport of HCO_3_^–^ lead to various diseases, including systemic acidosis, brain dysfunction, kidney stones, hyperammonaemia, hypertension, respiratory, gastrointestinal, and genitourinary system diseases, cystic fibrosis (CF), xerostomia, pancreatitis, and infertility [1,13,14]. HCO_3_^–^ has been used for the treatment of sepsis, which is a life-threatening medical emergency, and CF [19]. HCO_3_^–^ has been shown to improve the outcome of sepsis patients with acidosis [19]. Patients with renal tubular acidosis and diarrhoea are given HCO_3_^–^ replacement therapy. When HCO_3_^–^ is administered, it primarily increases plasma HCO_3_^–^ levels, thus countering acidaemia and helping restore the physiological pH balance [20]. The therapeutic effects of HCO_3_^–^ administration are especially important in conditions such as metabolic acidosis, where there is an imbalance in the acid-base equilibrium [20]. However, HCO_3_^–^ administration has several possible adverse effects. Some of these effects include an increased risk of hypokalemia, in which blood potassium levels become dangerously low [20]. There is also the possibility of inducing metabolic alkalosis, a condition in which there is a primary increase in serum HCO_3_^–^ [20].

The potential physiological consequences of administered HCO_3_^–^ might also include a sudden increase in haemoglobin-oxygen affinity through the Bohr effect, hypercapnia, and other related effects [21]. It has been shown that 89.3–100 mm HCO_3_^–^ is safe for the human body and has no considerable side effects. Treatment with intravenous infusion of HCO_3_^–^ leads to significant inhibition of lower respiratory tract pathogens such as bacteria, fungi, and mycobacteria. A cohort study regarding the oral rinse of sodium bicarbonate revealed a significant increase in salivary pH and prevented overgrowth of acid uric bacteria [22].

### HCO_3_^–^ and bacteria as foes: antimicrobial potential of HCO_3_^–^ and its influence on other antimicrobial agents

#### Antifungal and antibacterial activity of HCO_3_^–^

HCO_3_^–^ exhibits significant antibacterial and antifungal properties and can effectively inhibit the growth of *Streptococcus mutans*, especially when combined with sodium dodecyl sulphate [23]. A study on the antifungal activity of HCO_3_^–^ involving 70 fungal strains isolated from skin and nail infections revealed that HCO_3_^–^ (119.05 mm) could curb the growth of 80% of these pathogens. The effectiveness varies based on the type of fungi, with a minimum inhibitory concentration (MIC_90_) against yeast of 59.52 mm, dermatophytes requiring 238.10 mm, and moulds needing up to 476.19 mm [24]. Furthermore, when tested against clinical isolates, HCO_3_^–^ (119.05 mm) completely stopped the growth of 19 out of 24 samples and notably reduced the growth of the remaining samples within a week compared with samples without HCO_3_^–^ [24]. These findings suggest that HCO_3_^–^ can be used in combination with other antifungal agents, particularly for skin fungal infections and onychomycosis.

A recent study on the antibacterial properties of HCO_3_^–^ against *S. aureus*, *P. aeruginosa*, and *E. coli* has indicated its potential for the treatment of concomitant sepsis [25]. They reported a significant decrease in bacterial colonies within 24 h of HCO_3_^–^ exposure. Given that HCO_3_^–^ is already used to manage metabolic acidosis in intensive care units, these findings suggest that it may also offer antimicrobial benefits for patients with sepsis [25].

Recent studies have expanded our understanding of the antimicrobial and antibiofilm activities of HCO_3_^–^. It inhibits the growth and biofilm formation of various microbes, including *P. aeruginosa*, *Klebsiella pneumoniae* (*K. pneumoniae*), *Actinomyces naeslundii* (*A. naeslundii*), and *Candida albicans* (*C. albicans*). It also has a lethal effect on *Aspergillus parasiticus*, unrelated to pH or aflatoxin distribution [26]. HCO_3_^–^ drastically reduced aerobic plate counts in tests against aerobic and anaerobic bacteria such as *E. coli*, *S. aureus*, and *P. aeruginosa*. A 1,000-fold reduction was observed at a 120 mm concentration. Even common yeasts such as *S. cerevisiae* and *H. wingei* are highly sensitive, with counts reduced 100,000-fold by 60 mm HCO_3_^–^ [27].

Another study revealed that 1 M HCO_3_^–^ significantly inhibited bacterial, fungal, and mycobacterial growth both *in vitro* and *in vivo*. In both experiments, compared with saline (negative control), HCO_3_^–^ substantially reduced the number of colony-forming units for bacteria and fungi. Moreover, HCO_3_^–^ was particularly effective against *M. tuberculosis*, resulting in a lower prevalence of acid-fast bacilli than saline in both settings [28]. These findings highlight the potential of HCO_3_^–^ as an antimicrobial agent against a range of bacterial and fungal pathogens. Table 1 summarizes the antibacterial and antifungal activities of HCO_3_^–^.

**Table 1.** Antibacterial and antifungal activities of HCO_3_^–^.

Pathogen	Inhibition	Biofilm	Reference
*S. aureus*	+	+	[6,25,27],
*P. aeruginosa*	+	+	[6,25,27,29]
*E. coli*	+	–	[25,27]
*H. influenzae*	+	–	[29]
*B. cepacia*	+	–	[29]
*Prevotella intermedia*	+	–	[30]
*Streptococcus sanguinis*	+	–	[30]
*Aggregatibacter actinomycetemcomitans*	+	–	[4,30]
*Actinomyces viscosus*	+	–	[30]
*K. pneumoniae*	+	+	[26]
*A. naeslundii*	+	+	[26]
*Haemophilus aphrophilus* (*H. aphrophilus*)	+	–	[4]
*Eikenella corrodens* (*E. corrodens*)	+	–	[4]
*Capnocytophaga gingivalis* (*C. gingivalis*)	+	–	[4]
*M. tuberculosis*	+	–	[28]
*H. wingei*	+	–	[27]
*C. albicans*	+	+	[26]
*A. parasiticus*	+	–	[31]
*S. cerevisiae*	+	–	[24,27]

“+” indicates growth inhibition of the organism and inhibition of biofilm, whereas “–” indicates no growth inhibition or biofilm.

#### HCO_3_^–^ enhances antibacterial peptide activity

In human systems, the role of antimicrobial peptides (AMPs) as natural antibiotics, which often have no effect on microbes under culture conditions, remains largely elusive [7]. HCO_3_^–^ plays a key role in enhancing the effectiveness of AMPs, such as cathelicidins and defensins [32,33]. To test the hypothesis that HCO_3_^–^ enhances the effectiveness of AMPs, an *in vitro* study was conducted using animal cell cultures with HCO_3_^–^ in the medium. A study revealed that pathogenic bacteria, such as *S. aureus* and *E. coli*, changed cell wall thickness and sigma factor B expression, increasing their susceptibility to the AMP LL-37. These findings suggest that HCO_3_^–^ could act as a cofactor to increase the antimicrobial potency of AMPs, such as LL-37 [7]. Moreover, HCO_3_^–^ enhances the antimicrobial activity of various structurally different AMPs, including murine cathelicidins (mCRAM), linear porcine cathelicidin (PR-39), and both β-murine and human defensins (Cryptdin-4 and HBD-2) [7]. These findings shed light on the interplay between HCO_3_^–^ and AMPs and suggest that HCO_3_^–^ can act as a cofactor to increase the antimicrobial potency of AMPs.

HCO_3_^–^ is a crucial component of the buffering system in the human body and has a broad-spectrum antimicrobial effect. It also amplifies the antibacterial activity of other innate immune elements. A previous study investigated the effects of physiological concentrations of HCO_3_^–^ (25 mm) on various innate immunity mediators, including defensins and cathelicidins [34]. A previous study revealed that HCO_3_^–^ enhanced the antimicrobial activities of α-defensin, LL-37, indolicidin, bactenesin, and leukocyte protegrin against *E. coli* and *S. aureus* [34].

Moreover, many innate immune system components can disrupt the bacterial membrane potential. This suggests that the host uses a coordinated approach to target bacterial PMF in an HCO_3_^–^-rich environment [35]. Thus, HCO_3_^–^ possesses intrinsic antibacterial properties and is vital for boosting the immune response. It synergizes with the body’s physical and chemical defences to effectively eliminate infection-causing pathogens [35].

#### Effect of HCO_3_^–^ on other antimicrobial compounds

Innovative antibacterial drug screening methods that mimic the host environment where bacteria reside continue to emerge [36]. Recent advances involve chemicals specifically designed to inhibit bacterial growth under conditions that closely mimic those within the host [35]. HCO_3_^–^ is a chemical that is gaining attention for its ability to augment the activity of antimicrobial agents [37]. For example, a previous study demonstrated a significant synergistic effect when HCO_3_^–^ (5 mm) was combined with kanamycin (3.12 µg/mL), resulting in an 80% (w/v) reduction in *E. coli* growth. In contrast, when used alone, HCO_3_^–^ and kanamycin reduce *E. coli* growth by only 5% (w/v) and 15% (w/v), respectively [37]. Interestingly, HCO_3_^–^ also displayed paradoxical eagle-like behaviour at concentrations greater than 20 mm. Further studies are needed to understand its variable interactions with antibiotics, as antibiotic activity is enhanced and suppressed depending on antibiotic concentration [37].

Studies with 25 mm HCO_3_^–^ revealed its ability to increase the efficacy of eight antibiotic classes against both gram-positive and gram-negative bacteria [35]. Specific antibiotics, such as fluoroquinolones, tetracyclines, fosfomycin, and novobiocin, showed variable responses depending on the HCO_3_^–^ concentration. This synergistic activity was not pH dependent but was rooted in the chemical properties of HCO_3_^–^.

One study reported that HCO_3_^–^ disrupts the proton motive force (PMF) in bacterial cells, thereby affecting antibiotic effectiveness. The PMF, which is crucial for bacterial energy production, consists of an electrical potential (Δψ) and a proton gradient (ΔpH) [38]. The influence of HCO_3_^–^ varies depending on the antibiotic and its reliance on the PMF components. For example, it suppresses the use of tetracyclines, which rely on the ΔpH, while enhancing the use of aminoglycosides, which depend on the Δψ [39,40]. This suggests that HCO_3_^–^ modulates antibiotic uptake by altering PMF components, with effects extending to fluoroquinolones, depending on the specific conditions (Table 2) [35].

**Table 2.** Effect of HCO_3_^–^ on antibiotic activity.

Antibiotic	Activity of HCO_3_^–^	Reference
Gentamicin, kanamycin, fluoroquinolones, macrolides aminoglycosides, polymyxin B, tobramycin ß-lactam, cefazolin (CFZ), and oxacillin (OXA)	Synergistic	[12,37,41,42]
Tetracyclines, fosfomycin, novobiocin, some fluoroquinolones such as delafloxacin, kanamycina, gentamicina, nigericin, tobramycina	Antagonistic	[12,35,41]

^a^HCO_3_^–^ concentration >20 mm had an antagonistic effect on kanamycin.

**Figure 1.**
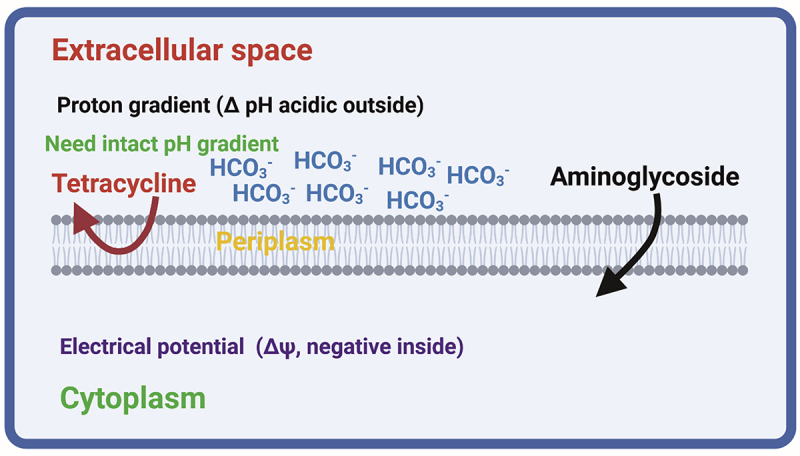
A model depicts the effect of HCO_3_^–^ on various classes of antibiotics. Credit: created via BioRender.com.

Figure 1 illustrates a hypothetical model that shows the influence of HCO_3_^–^ on antibiotics with different physicochemical properties. HCO_3_^–^ is a constituent of the medium ΔpH, representing the acidic extracellular environment and contributing to the PMF across the cytoplasmic membrane in gram-negative and gram-positive bacterial species. Aminoglycosides with a positive charge utilize the negative interior component of the membrane potential (Δψ) for transportation [35].

One study explored through transcriptional analysis how HCO_3_^–^ enhances the efficacy of aminoglycosides in *E. coli* [37,43]. In this study, HCO_3_^–^ (40 mm) treatment for 40 min during the logarithmic growth phase reduced the growth of viable *E. coli*. Using RNA-seq analysis, the study revealed that HCO_3_^–^ significantly altered gene expression, resulting in a tenfold increase in the expression of *tnaA*, which encodes tryptophanase, an enzyme crucial for tryptophan degradation. These results imply that HCO_3_^–^ could influence bacterial metabolism and susceptibility to antibiotics, although the specific mechanism remains unclear. This study highlights the complex interactions between HCO_3_^–^ and bacterial growth. Elevated expression of *tnaA*, which leads to increased indole production, was found to work synergistically with HCO_3_^–^ to inhibit the growth of *E. coli*. These findings suggest a potential role for indoles in growth inhibition. Moreover, HCO_3_^–^ led to significant changes in iron metabolism, as indicated by the upregulation of genes related to iron acquisition and the downregulation of genes related to iron – sulphur proteins. These findings suggest that HCO_3_^–^-induced growth inhibition may be partially due to iron deficiency [37,43]. In another study, HCO_3_^–^ was found to have varying effects on the effectiveness of fluoroquinolone antibiotics (Table 2) [41]. Specifically, it prevents the intracellular accumulation of delafloxacin, reducing its efficacy against multidrug-resistant *S. aureus* and *P. aeruginosa*.

Interestingly, HCO_3_^–^ also exhibited dual behaviour with tobramycin, first showing increased antibiotic efficacy against planktonic *P. aeruginosa* but having an antagonistic effect that encouraged biofilm growth. These findings highlight the nuanced roles that HCO_3_^–^ can play in modulating antibiotic activity and underscore the importance of understanding these interactions for more effective treatment strategies [12].

The increasing threat of antibiotic-resistant bacteria necessitates a comprehensive and globally coordinated response to ensure the continued efficacy of antibiotic treatments. The reliance of the healthcare industry on a single type of laboratory test for antibiotic susceptibility is a significant shortcoming, especially given that this test often does not replicate the complex interplay between hosts and pathogens in a living system. Emerging research has highlighted the limitations of standard antimicrobial susceptibility testing (AST) conducted in laboratory-specific media, which can produce misleading results. Some antibiotics that have proven ineffective in standard tests are highly effective in media that mimic host conditions. Conversely, certain antibiotics that pass conventional AST have demonstrated poor performance in living organisms.

A promising avenue for improving AST predictive accuracy involves incorporating HCO_3_^–^ into the test medium. HCO_3_^–^, a common molecule in biological systems, triggers far-reaching changes in bacterial physiology and gene expression. By better simulating *in vivo* conditions, this modification enhances the ability of the test to identify effective treatments, thereby streamlining the development and prescription of antibiotics. The incorporation of HCO_3_^–^ and other host-specific conditions into AST protocols could revolutionize the approach to combat antibiotic-resistant bacteria. By moving towards a more nuanced and biologically accurate model of infection, these advancements could lead to more targeted and effective therapies, reducing the risk of antibiotic resistance and improving patient outcomes [44].

## HCO_3_^–^ sensitizes MRSA to β-lactams by regulating gene expression

There are two types of methicillin-resistant *S. aureus* (MRSA): one is susceptible to β-lactams, such as oxacillin and cefazolin, in the presence of HCO_3_^–^, whereas the other is not [45]. The effect of HCO_3_^–^ on susceptibility is multifaceted: it alters PMF and downregulates key resistance genes, *mecA*, and *sarA*, in responsive strains [35,45,46]. Studies indicate that HCO_3_^–^ in culture media enhances the susceptibility of specific MRSA strains to β-lactams, such as cefazolin and oxacillin. Enhanced susceptibility was also observed in *ex vivo* endocarditis models, where HCO_3_^–^sensitizes MRSA strains to β-lactams. This effect varies depending on the genetic background of the strain [42,45–47].

**Figure 2.**
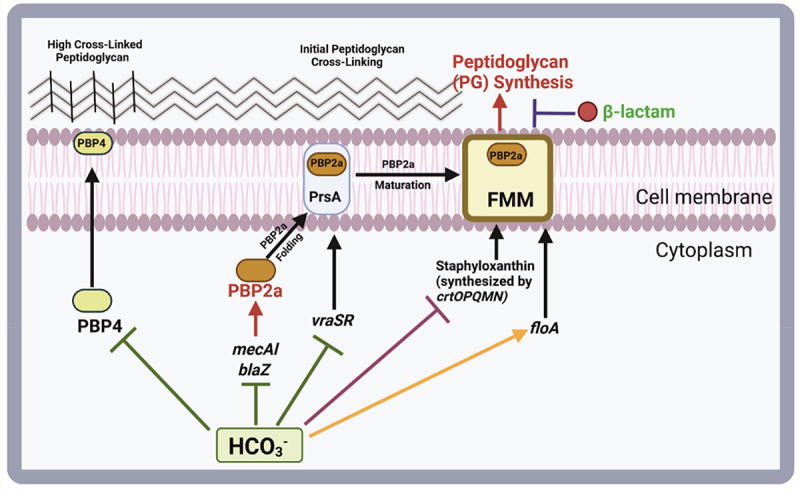
The model illustrates the effects of HCO_3_^–^ on PBP2 and peptidoglycan biosynthesis. HCO_3_^–^responsive strains display effects such as decreased production of the PBP2a protein, reduced expression of *pbp4* and *vraSR*, and lower levels of *PrsA*. Moreover, HCO_3_^–^ inhibits carotenoid production and increases *floA* expression, leading to unstable functional membrane microdomains (FMMs) [46]. Credit: created via BioRender.com.

HCO_3_^–^ exposure modulates gene expression in MRSA strains, affecting key genes, such as *mecA, blaZ, pbp4, vraSR, prsA, sigB*, and *floA*, which are critical for alternative penicillin-binding protein (PBP2a) production and maturation and membrane PBP2a and PrsA protein content. Specifically, it significantly downregulated *mecA, blaZ, the vraSR-prsA gene axis, pbp4*, and carotenoids (Figure 2) while upregulating *floA* across all MRSA strains [46].

These findings revealed that HCO_3_^–^ modulates a set of genes essential for the HCO_3_^–^-responsive MRSA phenotype via PBP2a function and maturation [46]. Another study revealed that HCO_3_^–^-responsive MRSA isolates are susceptible to CFZ and OXA, and genotypic markers such as clonal complex 8 [CC8], agr I, and spa t008 are associated with ^τηε^ responsiveness of HCO_3_^–^to OXA [42]. RNA-Seq identified key genes that were differentially expressed in HCO_3_^–^-responsive strains, including those in the sigB-sarA-agr axis, cell wall-associated genes, and those related to autolysis (Table 3) [46]. Studies with a fluorescent penicillin probe (bocillin-FL) suggested that HCO_3_^–^ affects β-lactam binding to both the cell surface and PBP2a, indicating the responsiveness of MRSA to β-lactams [47].

**Table 3.** Regulation of genes by HCO_3_^–^ and susceptibility to β-lactams.

Regulated genes	Pathogen	Pathway effected	Reference
*mecA and sarA*	MRSA HCO_3_^–^-responsive	Production/maturation of PBP2a and PrsA protein	[42]
*crtM, sigB, sarA, agrA, hla, fnbA, and icaA*	*S. aureus*	Regulation of virulence factors	[48]
*mecA and blaZ, vraSR-prsA gene axis, and pbp4.*	MRSA HCO_3_^–^-responsive	Production/maturation of PBP2a,	[49]
*sigB-sarA-agr regulon cap8, clpL, sasD, aaa, vra X,kdpABCDF, betAB, icaR, rsp, clfA, clfB,agr, sdrH,fnbA, fnbB, atl, sceD, isaA, fmtA, ddh, pbp2, bccT,usp*	MRSA HCO_3_^–^-responsive and nonresponsive	Virulence autolysins cell wall synthesis, osmotic stress response,	[46]

**Figure 3.**
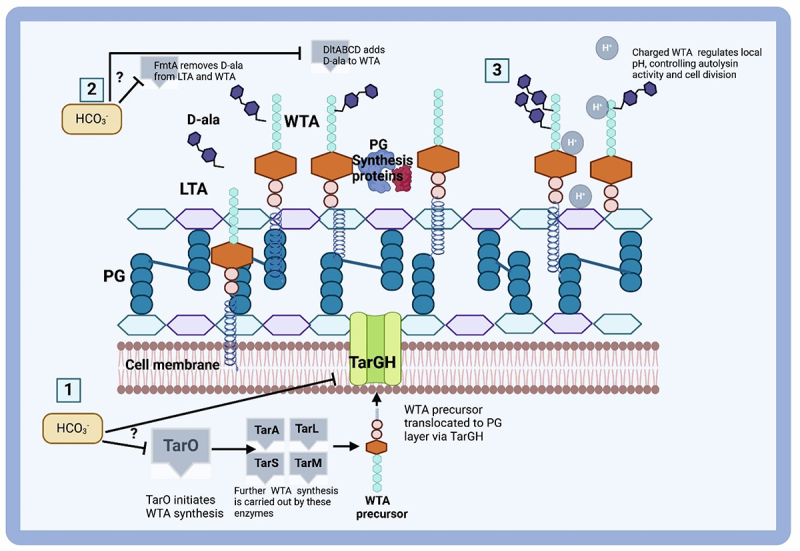
The model elucidates the impact of HCO_3_^–^ on the synthesis of WTA and the susceptibility of strains responsive to β-lactams [50]. Credit: created via BioRender.com.

Recent research revealed that HCO_3_^–^ decreases wall teichoic acid (WTA) levels and molecular weights in HCO_3_^–^-responsive MRSA strains. It also induces increased autolysis and irregular cell division, both of which are associated with the disruption of WTA synthesis. These data suggest that HCO_3_^–^ inhibits WTA biosynthesis via a posttranslational mechanism involving specific genes such as *tarO, tarG, dltA*, and *fmtA* (Figure 3). This study revealed that HCO_3_^–^ directly influences WTA biosynthesis in HCO_3_^–^-responsive MRSA strains [50].

Figure 3 shows the pathways involved in the synthesis and maturation of PG and WTA, underscoring the role of HCO_3_^–^ in affecting these processes and contributing to the β-lactam- and HCO_3_^–^-responsive phenotype. This study confirms the inhibitory effect of HCO_3_^–^ on WTA production, which is linked to the increased susceptibility of MRSA to β-lactam antibiotics [50]. Another study revealed that HCO_3_^–^ enhances the efficacy of antibacterial drugs against *S. aureus* both *in vitro* and *in vivo*, reducing the production of virulence factors and increasing susceptibility to oxidative stress [48].

## HCO_3_^–^ and bacteria as friends: regulation of virulence genes in pathogenic strains

### Effect of HCO_3_^–^ on virulence expression in *E. coli*

Enterohemorrhagic *E. coli* (EHEC), known for causing bloody diarrhoea and haemolytic uraemic syndrome, adheres to the intestinal mucosa and creates attaching and effacing (A/E) lesions via the locus for enterocyte effacement (LEE) genes. Studies have shown that HCO_3_^–^ in the medium increases bacterial adherence and the expression of LEE-encoded genes, such as *intimin, Tir, EspA*, and *EspB* [51]. Furthermore, the expression of *ler*, a crucial regulator of LEE-encoded genes, depends on the HCO_3_^–^ concentration in the medium. This suggests that HCO_3_^–^ acts as an intestinal signalling molecule, facilitating EHEC colonization, particularly in the lower intestine, where the HCO_3_^–^ concentration is relatively high [51].

**Figure 4.**
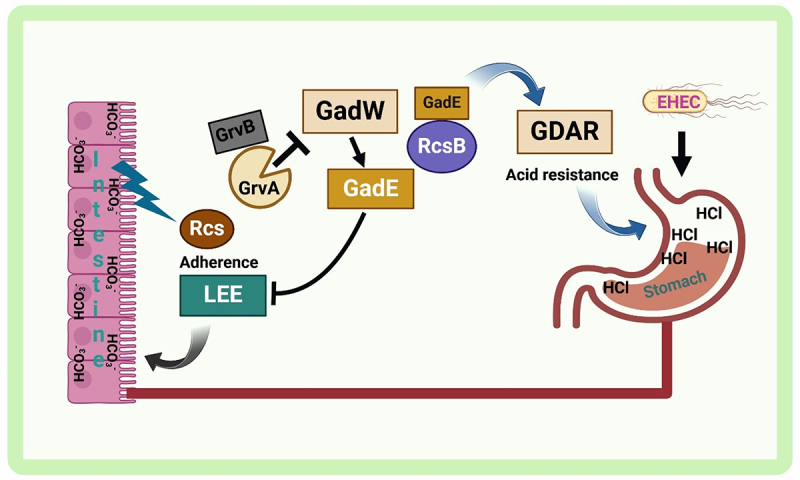
The model explains the GrvA-dependent regulation of acid resistance (GDAR) and lee-dependent adherence by *E. coli*. Credit: created via BioRender.com.

Another study has shown that HCO_3_^–^ is crucial for activating the *rcsDB* and *rcsB* genes. An intact Rcs system and grvA activator are also vital for LEE stimulation, highlighting the role of HCO_3_^–^ in the virulence of intestinal pathogens [52]. Notably, *RcsB* activates and represses LEE transcription and requires HCO_3_^–^ for activation [53]. Both *rcsB* and *GrvA* are essential for this HCO_3_^–^-induced activation [52]. *GrvA* and *RcsB* jointly mediate the role of HCO_3_^–^ in activating the LEE pathway, which is critical for intestinal pathogen colonization (Figure 4).

### Effect of HCO_3_^–^ on virulence expression in *Vibrio cholerae*

*V. cholerae*, a gram-negative bacterium, causes cholera, which is characterized by severe diarrhoea. The pathogen has two primary biotypes, classical and El Tor, each with unique *in vitro* growth conditions for virulence gene expression [54]. When exposed to specific triggers or upon infection, *V. cholerae* initiates a complex regulatory cascade. This leads to the production of the regulatory protein ToxT, which activates the transcription of key virulence genes, including those encoding cholera toxin (CT), toxin-coregulated pilus (TCP), and other important virulence genes [11].

**Figure 5.**
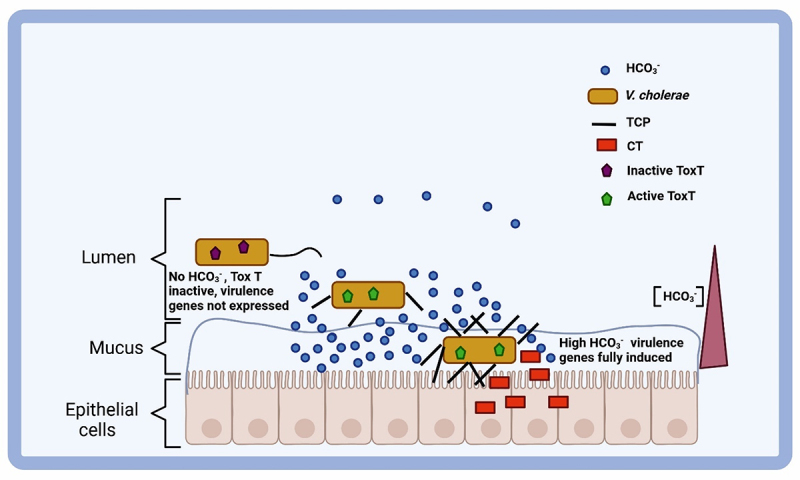
The model illustrates the expression of virulence genes by HCO_3_^–^. *V. cholerae* strains carrying inactive ToxT protein enter the upper small intestine (left). The HCO_3_^–^ in the intestinal lumen (center), ToxT, activates TCP production. In the mucus layer, higher HCO_3_^–^ levels (right) induce virulence genes and subsequent CT production. Credit: created via BioRender.com.

Studies have shown that HCO_3_^–^ stimulates CT expression in the *V. cholerae* El Tor biotype and enhances ToxT activity, a key regulatory protein for virulence genes [11]. Both the classical and El Tor biotypes showed inactive ToxT in experiments without HCO_3_^–^. However, adding HCO_3_^–^ significantly upregulated CT and TCP expression in both biotypes without altering ToxT production levels. The presence of ethoxzolamide, a CA inhibitor, disrupted this HCO_3_^–^-mediated virulence induction, suggesting the role of CO_2_ to HCO_3_^–^ conversion by CA in enhancing virulence [11]. HCO_3_^–^ is a key chemical trigger for virulence gene activation, as *V. cholerae* colonizes the HCO_3_^–^-rich upper small intestine (Figure 5) [55].

### Effect of HCO_3_^–^ on virulence expression in *Bacillus cereus* and *Bacillus anthracis*

*Bacillus cereus* (*B. cereus*) is a gram-positive, rod-shaped foodborne pathogen responsible for gastrointestinal symptoms and potentially fatal lower respiratory infections, even with antibiotic treatment [56,57]. Another member of this bacterial group, *Bacillus anthracis* (*B. anthracis*), is known to cause anthrax and is considered a bioterrorism agent because of its lethal nature and the plasmids p×O1and p×O2[58].

HCO_3_^–^ and CO_2_, which are essential for pH regulation in the body, increase the expression of genes associated with anthrax toxin components [59]. These components make the environment in mammalian hosts conducive to pathogenic bacteria that thrive in HCO_3_^–^-rich settings. Indeed, both CO_2_ and HCO_3_^–^ are vital to host‒pathogen dynamics, especially in *B. anthracis* [2,60].

The virulence plasmid p×O1harbors genes for significant anthrax toxins and poly γ-d-glutamic acid capsule (PGA), a key virulence factor [61,62]. PGA transcription is increased by CO_2_/HCO_3_^–^ through positive regulation of the capsule biosynthetic operon capBCAD and the plasmid regulators *atxA, acpA*, and *acpB* [63]. *In vitro* studies have demonstrated that genes controlled by *atxA*, *acpA*, and *acpB* are induced by HCO_3_^–^ [59,64,65]. A study has shown that in atxA 1 strains, elevated CO_2_/HCO_3_^–^ and temperature increase the expression of toxin genes, namely, *pag, lef*, and *cya* [61]. Interestingly, CO_2_/HCO_3_^–^ and temperature combined bolster toxin gene expression, with a sixfold increase in *atxA* mRNA expression at 37°C compared with that at 28°C [59]. These findings indicate that HCO_3_^–^ and temperature jointly regulate the expression of the three *B. anthracis* toxin genes in a coordinated manner [59].

Another study comparing the gene expression profiles of *B. cereus* strain G9241 and an attenuated *B. anthracis* (Sterne 34F 2) strain in high CO_2_/HCO_3_^–^ (1666.67 mm) environments versus ambient air identified marked differences in gene expression in the presence of CO_2_/HCO_3_^–^ [66,67]]. Intriguingly, gene expression in the G9241 strain differs from that in *B. anthracis*, possibly because it is regulated by PlcR and anthrax toxin activator (AtxA) transcriptional regulators [66,67].

### Effect of HCO_3_^–^ on virulence in *citrobacter rodentium* and *E. faecalis*

*C. rodentium* is a gram-negative bacterium that predominantly infects mouse intestines and occasionally acts as an opportunistic pathogen in humans [68]. A study focusing on how a SlyS-like regulator interacts with environmental factors, particularly HCO_3_^–^, revealed that HCO_3_^–^ activates the transcription of the *adcA* and *kfc* genes. These genes are essential for bacterial adhesion and colonization. This study revealed that HCO_3_^–^ triggers the transcription of these genes and enhances the binding affinity of RegA for its target DNA [2]. These findings highlight the significant role of HCO_3_^–^ in modulating bacterial virulence gene expression, potentially offering new avenues for understanding bacterial colonization and infection processes.

Antibiotic-resistant *E. faecalis* can cause urinary tract infections (UTIs), which present healthcare challenges. In *E. faecalis*, the gene *EbpR*, a member of the *AtxA/Mga* regulator family, influences biofilm development by increasing the expression of the endocarditis and biofilm-associated pilus operon (*ebpABC*) [69,70]. A study showed that treating *E. faecalis* with 100 mm HCO_3_^–^ upregulated *EbpR* and related genes and enhanced pili production and biofilm formation [69]. Research has revealed 73 HCO_3_^–^-responsive genes that are linked mainly to transport systems, indicating that HCO_3_^–^ modulates virulence in *E. faecalis* in a manner similar to its effects on other pathogens, such as *V. cholerae*, *B. anthracis*, *C. neoformans*, and *C. rodentium* [2,55,63,71,72].

### Role of HCO_3_^–^ in mycobacterial virulence gene expression

In nontuberculous mycobacteria (NTMs), HCO_3_^–^ has been shown to influence the export of extracellular DNA (eDNA), which is crucial for antibiotic resistance and biofilm formation, and this influence is independent of pH (Table 4) [73]. Interestingly, in *M. avium*, inactivation of CAs diminishes the transport of eDNA, whereas re-establishment of CA activity restores eDNA transport. This highlights the significant role of CAs in the release of eDNA and biofilm formation [73]. Ethoxzolamide (ETZ), a potent CA inhibitor, significantly impairs eDNA export, suggesting that CA plays a role via HCO_3_^–^ [8,75].

**Table 4.** Regulation of genes by HCO_3_^–^ and pathways in pathogenic bacteria.

Pathogen	Genes/Component	Effect	Reference
*E. coli*	*rcsDB, rcsB, ler, Tir, EspA*, and *EspB,*	Toxin secretion and virulence	[51–53]
*V. cholerae*	CT, ToxT, TCP, *pag, lef*, and *cya*	Toxin secretion and virulence	[11]
*B. anthracis*	PGA capsule, *capBCAD, atxA, acpA, acpB, pag, lef*, and *cya*	Virulence, toxins	[11,61–63,71]
*C. neoformans*	Cac1adenylyl cyclase	Capsule synthesis	[72]
*E. faecalis*	*ebpR, ebpABC*	Virulence, biofilm	[69,70]
*C. rodentium*	*adcA, kfc, RegA*	Virulence	[2]
*M. avium*	eDNA	Biofilm formation	[73]
*M. tuberculosis*	Esx-1	Virulence	[74]

In addition, in *M. tuberculosis*, ETZ disrupts the PhoPR signalling pathway, resulting in changes similar to those observed in PhoPR mutants, such as downregulation of the PhoPR regulon, reduction in virulence-associated lipids, and inhibition of Esx-1 protein secretion (Table 4) [74]. This highlights a different aspect of the role of HCO_3_^–^ in bacterial physiology, contrasting its inhibition of gram-positive and gram-negative bacteria, including *M. tuberculosis*.

### Bicarbonate and CO_2_ as mediators of host immunity and the regulation of pathogenicity

HCO_3_^−^ is the second most abundant anion in the human body; in addition to its role in pH regulation, it controls the activity of many proteins in the body, including immune components [14]. In cystic fibrosis (CF), CF transmembrane regulator (CFTR), an ATP binding cassette, is required for HCO_3_^−^ secretion, and a mutation in CFTR leads to a defect in this anion in the CF lung. The bacteria found in CF are *S. aureus*, *H. influenzae* and *P. aeruginosa*, in which the host immune system responds via leukocytes and other immune components, including AMPs [76]. It has been demonstrated that the use of HCO_3_^−^ enhances this immune response to combat these pathogens in CFs both *ex vivo* and *in vivo* [76]. Furthermore, when combined with vaccines, HCO3- significantly increases the immune response in poultry, suggesting its importance in the immunity of the host [77]. In mice, inoculation of a vaccine with HCO_3_^−^ significantly enhances the protective immune response against *Brucella* compared with that of a formulation without HCO_3_^−^ [78].

An increase in CO_2_ or hypercapnia in multiple inflammatory diseases is known to suppress immune cell activity [79]. It has also been shown that bacterial infections and hypercapnic acidosis impair immune cell function, leading to immunosuppression and increased patient mortality after pneumonia [79,80]. A recent study demonstrated that elevated CO_2_ reduces monocyte and macrophage migration via inflammatory gene expression and decreases the intracellular pH, which is also dependent on the activity of CA, suggesting that CO_2_ immunomodulates immune cells through a CA2-coupled change in the intracellular pH [79]. In mice, increased CO_2_ altered the immune response to inflammatory agents such as lipopolysaccharide (LPS) and organic dust [81]. Recent studies carried out in mycobacteria revealed that high levels of CO_2_ induce PhoPR signalling, which is independent of pH, suggesting that the PhoPR regulon functions as a CO_2_ sensor. Interestingly, the CA inhibitor ethoxzolamide (ETZ) inhibited PhoPR signalling, supporting the hypothesis that CO_2_ plays a role in regulating PhoPR. Knockdown of CA resulted in a reduction in virulence. Transcriptional profiling studies at 5% CO_2_ revealed the induction of PhoPR regulon genes, which include the ESX-1 secretion system [82].

## CAs and their inhibition in bacteria as an antimicrobial approach

Pathogenic microbes have become resistant to clinically used drugs, and novel antimicrobial compounds that target novel pathways of these pathogens are needed [83,84]. In the recent past, sequencing the genomes of pathogenic microbes revealed many alternate pathways that are crucial for their life cycle and can be targeted via novel antimicrobials devoid of resistance [84]. Among the alternative pathways associated with these pathogens, carbonic anhydrases play crucial roles and have been used as targets for the development of antimicrobial agents via small-molecule inhibitors. Pathogenic organisms contain both α-CAs and β-CAs that have been cloned, and studies have shown that CAs play crucial roles in the survival and pathogenesis of pathogens [85].

Pathogens sense CO_2_ in their environment via HCO_3_^−,^ which is generated by the enzymatic activity of CAs and regulates the expression of genes required for the virulence of these microbes [84]. In addition, studies have demonstrated that CAs are involved in many other functions, such as biofilm formation and survival in the host environment. In *E. faecalis*, disruption of α-CA sensitized bacteria to killing with gentamicin [86]. In *P. aeruginosa*, β-CA is required for calcium deposition and contributes to virulence [4]. The inactivation and inhibition of the activity of this CA chemical inhibitor reduced calcium deposition in this bacterium [73]. In mycobacterial species, CAs are required to transport eDNA, a component of biofilms, and to express virulence factors. Inhibition via EZA or inactivation of CA has been shown to reduce biofilm formation and attenuate virulence [8,9,73,74]. The inhibition of β-CAs with a specific inhibitor significantly reduced the bacterial load *in vivo* in zebrafish larvae [75]. In addition, in *in vivo* studies involving *Neisseria* spp., *H. pylori, B. suis, and S. pneumoniae*, the growth of these pathogens could be impaired via the use of CA inhibitors [87]. *H. pylori* encounter bicarbonate, urea and acid in gastric environment. Analysis of *H. pylori* mutants showed that CAs play a role in maintaining activity of urease and acid resistance through HCO_3_^−^ in an acidic environment, suggesting the requirement of CAs for the bacterium for survival in the gastric niche [88].

The crystal structures of many microbial CAs have been resolved, and inhibition studies using different classes of inhibitors have been performed [85]. The CA inhibitors sulphonamides, dithiocarbamates, and inorganic anions have been shown to inhibit the alpha- and beta-CAs of bacteria and fungi. Among CA inhibitors, sulphonamides/sulfamates represent one of the main classes of CAIs [89]. These compounds are already in clinical use for the treatment of various diseases, including acetazolamide, methazolamide, and ethoxzolamide, which inhibit all CAs, including CAs, from pathogenic microbes. Sulphonamides/sulfamates/sulamides have been shown to inhibit CAs both *in vitro* and *in vivo* in mice infected with antibiotic-resistant strains, confirming their role in contrasting bacterial antibiotic resistance [90]. In addition, phenol inhibitors, either alone or in combination with clinically used antibiotics, inhibit the growth of the bacterium and biofilm formation [87]. Both *in vitro* and *in vivo* inhibition studies have shown that the CAs of these pathogens can be novel targets for combating microbial infections that are devoid of resistance [8,9,75,91,92].

## Concluding remarks

HCO_3_^–^ plays a pivotal role in human health and disease, and its role in cellular physiology involves pH regulation, suggesting its impact on various metabolic and signalling pathways. In the realm of microbial studies, HCO_3_^–^ has emerged as an agent of interest owing to its importance not only in microbial physiology but also in human physiology. The antimicrobial effects of HCO_3_^–^ are manifested in diverse ways, first by inhibition of the growth of pathogenic bacteria directly and second by increasing the effects of different antibiotics on pathogenic bacteria.

However, HCO_3_^–^ is a double-edged sword, as HCO_3_^–^ also regulates genes and increases virulence in certain pathogenic bacteria. This underlines the need for caution before integrating HCO_3_^–^ into clinical applications that target bacterial infections. As researchers dive deeper, the dichotomous nature of the effects of HCO_3_^–^, both inhibitory and promotional, on bacterial virulence becomes evident. The path ahead necessitates a thorough and nuanced understanding of HCO_3_^–^ before its widespread adoption in microbial therapies.

## Data Availability

The authors confirm that the data supporting the findings of this study are available within the article.

## References

[1] Aspatwar A, Tolvanen MEE, Barker H, et al. Carbonic anhydrases in metazoan model organisms: molecules, mechanisms, and physiology. Physiol Rev. 2022;102(3):1327–15. doi: 10.1152/physrev.00018.2021 35166161

[2] Yang J, Hart E, Tauschek M, et al. Bicarbonate-mediated transcriptional activation of divergent operons by the virulence regulatory protein, RegA, from citrobacter rodentium. Mol Microbiol. 2008;68(2):314–327. doi: 10.1111/j.1365-2958.2008.06171.x 18284589

[3] Griffiths E, Humphreys J. Bacteriostatic effect of human milk and bovine colostrum on Escherichia coli: importance of bicarbonate. Infect Immun. 1977;15(2):396–401. doi: 10.1128/iai.15.2.396-401.1977 14890 PMC421381

[4] Miyasaki KT, Genco RJ, Wilson ME. Antimicrobial properties of hydrogen peroxide and sodium bicarbonate individually and in combination against selected oral, gram-negative, facultative bacteria. J Dent Res. 1986;65(9):1142–1148. doi: 10.1177/00220345860650090601 3016051

[5] Corral LG, Post LS, Montville TJ. Antimicrobial activity of sodium bicarbonate. J Food Sci. 1988;53(3):981–982. doi: 10.1111/j.1365-2621.1988.tb09005.x

[6] Dobay O, Laub K, Stercz B, et al. Bicarbonate inhibits bacterial growth and biofilm formation of prevalent cystic fibrosis pathogens. Front Microbiol. 2018;9:2245. doi: 10.3389/fmicb.2018.02245 30283433 PMC6157313

[7] Dorschner RA, Lopez‐Garcia B, Peschel A, et al. The mammalian ionic environment dictates microbial susceptibility to antimicrobial defense peptides. FASEB J. 2006;20(1):35–42. doi: 10.1096/fj.05-4406com 16394265

[8] Aspatwar A, Kairys V, Rala S, et al. Mycobacterium tuberculosis β-carbonic Anhydrases: novel targets for developing antituberculosis drugs. Int J Mol Sci. 2019;20(20):20(20. doi: 10.3390/ijms20205153 PMC6834203 31627429

[9] Aspatwar A, Winum J-Y, Carta F, et al. Carbonic anhydrase inhibitors as novel drugs against mycobacterial β-carbonic anhydrases: an update on in vitro and in vivo studies. Molecules. 2018;23(11):2911. doi: 10.3390/molecules23112911 30413024 PMC6278287

[10] Jones RT, Talley RS. Effects of gaseous CO2 and bicarbonate on the growth of Neisseria gonorrhoeae. J Clin Microbiol. 1977;5(4):427–432. doi: 10.1128/jcm.5.4.427-432.1977 404318 PMC274618

[11] Abuaita BH, Withey JH. Bicarbonate induces vibrio cholerae virulence gene expression by enhancing ToxT activity. Infect Immun. 2009;77(9):4111–4120. doi: 10.1128/IAI.00409-09 19564378 PMC2738005

[12] Kaushik KS, Stolhandske J, Shindell O, et al. Tobramycin and bicarbonate synergise to kill planktonic Pseudomonas aeruginosa, but antagonise to promote biofilm survival. NPJ Biofilms Microbiomes. 2016;2(1):16006. doi: 10.1038/npjbiofilms.2016.6 28721244 PMC5515257

[13] Aspatwar A, Supuran CT, Waheed A, et al. Mitochondrial carbonic anhydrase VA and VB: properties and roles in health and disease. J Physiol. 2023;601(2):257–274. doi: 10.1113/JP283579 36464834 PMC10107955

[14] Shin DH, Kim M, Kim Y, et al. Bicarbonate permeation through anion channels: its role in health and disease. Pflugers Arch - Eur J Physiol. 2020;472(8):1003–1018. doi: 10.1007/s00424-020-02425-x 32621085

[15] Dobre M, Gaussoin SA, Bates JT, et al. Serum bicarbonate concentration and cognitive function in hypertensive adults. Clin J Am Soc Nephrol. 2018;13(4):596–603. doi: 10.2215/CJN.07050717 29567858 PMC5968905

[16] Delgado-Bermúdez A, Yeste M, Bonet S, et al. A review on the role of bicarbonate and proton transporters during sperm capacitation in mammals. Int J Mol Sci. 2022;23(11):23(11. doi: 10.3390/ijms23116333 PMC9180951 35683013

[17] Wang HS, Chen, Y., Vairamani, K. and Shull, G.E., Critical role of bicarbonate and bicarbonate transporters in cardiac function. World J Biol Chem. 2014;5(3):334–345. doi: 10.4331/wjbc.v5.i3.334 25225601 PMC4160527

[18] Wang T, Zhang Y, Zhang R, et al. Efficacy of nasal irrigation and oral rinse with sodium bicarbonate solution on virus clearance for COVID-19 patients. Front Public Health. 2023;11:1145669. doi: 10.3389/fpubh.2023.1145669 37006571 PMC10053493

[19] Velissaris D, Karamouzos V, Ktenopoulos N, et al. The use of sodium bicarbonate in the treatment of acidosis in sepsis: a literature update on a long Term debate. Crit Care Res Pract. 2015;2015:1–7. doi: 10.1155/2015/605830 PMC4534594 26294968

[20] Tanios BY, Omran MO, Noujeim C, et al. Carbonic anhydrase inhibitors in patients with respiratory failure and metabolic alkalosis: a systematic review and meta-analysis of randomized controlled trials. Crit Care. 2018;22(1):275. doi: 10.1186/s13054-018-2207-6 30371345 PMC6205780

[21] Coppola S, Caccioppola A, Froio S, et al. Sodium bicarbonate in different critically Ill conditions: from physiology to clinical practice. Anesthesiology. 2021;134(5):774–783. doi: 10.1097/ALN.0000000000003733 33721887

[22] Chandel S, Khan M, Singh N, et al. The effect of sodium bicarbonate oral rinse on salivary pH and oral microflora: a prospective cohort study. Natl J Maxillofac Surg. 2017;8(2):106–109. doi: 10.4103/njms.NJMS_36_17 29386812 PMC5773983

[23] Drake D. Antibacterial activity of baking soda. Compend Contin Educ Dent Suppl. 1996;17(19):S17–21. 11524862

[24] Letscher-Bru V, Obszynski CM, Samsoen M, et al. Antifungal activity of sodium bicarbonate against fungal agents causing superficial infections. Mycopathologia. 2013;175(1–2):153–158. doi: 10.1007/s11046-012-9583-2 22991095

[25] Kesici U, Kesici S, Demirci M. Bicarbonate may alters bacterial susceptibility to antibiotics by targeting Pseudomonas aeruginosa, Escherichia coli and staphylococcus aureus. J Contemp Med. 2019;9(3):245–248. doi: 10.16899/jcm.599259

[26] Gawande PV, LoVetri K, Yakandawala N, et al. Antibiofilm activity of sodium bicarbonate, sodium metaperiodate and SDS combination against dental unit waterline-associated bacteria and yeast. J Appl Microbiol. 2008;105(4):986–992. doi: 10.1111/j.1365-2672.2008.03823.x 18422552

[27] Corral LG, Post LS, Montville TJ. Antimicrobial activity of sodium bicarbonate: a research note. J Food Sci. 1988;53(3):981–982. doi: 10.1111/j.1365-2621.1988.tb09005.x

[28] Abdalla DA, El Badrawy M, Abou Elela M, et al. Effect of bronchoalveolar lavage with sodium bicarbonate on lower respiratory tract pathogens. Chest. 2016;149(4):149. doi: 10.1016/j.chest.2016.02.093

[29] Jaikumpun P, Ruksakiet K, Stercz B, et al. Antibacterial effects of bicarbonate in media modified to mimic cystic fibrosis sputum. Int J Mol Sci. 2020;21(22):21(22. doi: 10.3390/ijms21228614 PMC7696793 33207565

[30] Pratten J, Wiecek J, Mordan N, et al. Physical disruption of oral biofilms by sodium bicarbonate: an in vitro study. Int J Dent Hyg. 2016;14(3):209–214. doi: 10.1111/idh.12162 26198308

[31] Montville TJ, Goldstein PK. Sodium bicarbonate reduces viability and alters aflatoxin distribution of aspergillus parasiticus in Czapek’s agar. Appl Environ Microbiol. 1987;53(10):2303–2307. doi: 10.1128/aem.53.10.2303-2307.1987 2827567 PMC204104

[32] Nizet V, Ohtake T, Lauth X, et al. Innate antimicrobial peptide protects the skin from invasive bacterial infection. Nature. 2001;414(6862):454–457. doi: 10.1038/35106587 11719807

[33] Lee PH, Ohtake T, Zaiou M, et al. Expression of an additional cathelicidin antimicrobial peptide protects against bacterial skin infection. Proc Natl Acad Sci U S A. 2005;102(10):3750–3755. doi: 10.1073/pnas.0500268102 15728389 PMC549293

[34] Zasloff M. Antimicrobial peptides in health and disease. N Engl J Med. 2002;347(15):1199–1200. doi: 10.1056/NEJMe020106 12374882

[35] Farha MA, French S, Stokes JM, et al. Bicarbonate alters bacterial susceptibility to antibiotics by targeting the proton motive force. ACS Infect Dis. 2018;4(3):382–390. doi: 10.1021/acsinfecdis.7b00194 29264917

[36] Farha MA, Brown ED. Unconventional screening approaches for antibiotic discovery. Ann N Y Acad Sci. 2015;1354(1):54–66. doi: 10.1111/nyas.12803 26100135

[37] Gutiérrez-Huante M, Martínez H, Bustamante VH, et al. Bicarbonate enhances the in vitro antibiotic activity of kanamycin in Escherichia coli. Lett Appl Microbiol. 2015;60(5):440–446. doi: 10.1111/lam.12388 25585891

[38] Bakker EP, Mangerich WE. Interconversion of components of the bacterial proton motive force by electrogenic potassium transport. J Bacteriol. 1981;147(3):820–826. doi: 10.1128/jb.147.3.820-826.1981 6268609 PMC216117

[39] Yamaguchi A, Ohmori H, Kaneko-Ohdera M, et al. Delta pH-dependent accumulation of tetracycline in Escherichia coli. Antimicrob Agents Chemother. 1991;35(1):53–56. doi: 10.1128/AAC.35.1.53 2014981 PMC244940

[40] Taber HW, Mueller JP, Miller PF, et al. Bacterial uptake of aminoglycoside antibiotics. Microbiol Rev. 1987;51(4):439–457. doi: 10.1128/mr.51.4.439-457.1987 3325794 PMC373126

[41] Holland M, Bjanes E, Nizet V, et al. Bicarbonate modulates delafloxacin activity against MDR staphylococcus aureus and Pseudomonas aeruginosa. J Antimicrob Chemother. 2022;77(2):433–442. doi: 10.1093/jac/dkab421 34893834 PMC8809187

[42] Rose WE, Bienvenida AM, Xiong YQ, et al. Ability of bicarbonate supplementation to sensitize selected methicillin-resistant staphylococcus aureus strains to β-lactam antibiotics in an ex vivo simulated endocardial vegetation Model. Antimicrob Agents Chemother. 2020;64(3). doi: 10.1128/AAC.02072-19 PMC7038310 31844004

[43] Gutiérrez-Huante M, Martínez‐Duncker ME, Sauceda E, et al. The antibiotics potentiator bicarbonate causes upregulation of tryptophanase and iron acquisition proteins in Escherichia coli. Lett Appl Microbiol. 2019;68(1):87–95. doi: 10.1111/lam.13092 30382577

[44] Ersoy SC, Heithoff DM, Barnes L, et al. Correcting a fundamental flaw in the paradigm for antimicrobial susceptibility testing. EBioMedicine. 2017;20:173–181. doi: 10.1016/j.ebiom.2017.05.026 28579300 PMC5478264

[45] Ersoy SC, Abdelhady W, Li L, et al. Bicarbonate resensitization of methicillin-resistant staphylococcus aureus to β-lactam antibiotics. Antimicrob Agents Chemother. 2019;63(7):63(7. doi: 10.1128/AAC.00496-19 PMC6591647 31010857

[46] Ersoy SC, Hanson BM, Proctor RA, et al. Impact of bicarbonate-β-lactam exposures on methicillin-resistant staphylococcus aureus (MRSA) gene expression in bicarbonate-β-lactam-Responsive vs. Non-Responsive strains. Genes (Basel). 2021;12(11):1650. doi: 10.3390/genes12111650 34828256 PMC8619011

[47] Ersoy SC, Chan LC, Yeaman MR, et al. Impacts of NaHCO3 on β-lactam binding to PBP2a protein variants associated with the NaHCO3-responsive versus NaHCO3-non-responsive phenotypes. Antibiotics (Basel). 2022;11(4):11(4. doi: 10.3390/antibiotics11040462 PMC9028190 35453214

[48] Saleh MM, Yousef N, Shafik SM, et al. Attenuating the virulence of the resistant superbug Staphylococcus aureus bacteria isolated from neonatal sepsis by ascorbic acid, dexamethasone, and sodium bicarbonate. BMC Microbiol. 2022;22(1):268. doi: 10.1186/s12866-022-02684-x 36348266 PMC9644464

[49] Ersoy SC, Chambers HF, Proctor RA, et al. Impact of bicarbonate on PBP2a production, maturation, and functionality in methicillin-resistant Staphylococcus aureus (MRSA). Antimicrob Agents Chemother. 2023;65(5):65(5. doi: 10.1128/AAC.02621-20 PMC8092911 33649115

[50] Ersoy SC, Gonçalves B, Cavaco G, et al. Influence of sodium bicarbonate on Wall Teichoic acid synthesis and β-lactam sensitization in NaHCO 3 -responsive and nonresponsive methicillin-resistant staphylococcus aureus. Microbiol Spectr. 2022;10(6):e0342222. doi: 10.1128/spectrum.03422-22 36377886 PMC9769754

[51] Abe H, Tatsuno I, Tobe T, et al. Bicarbonate ion stimulates the expression of locus of enterocyte effacement-encoded genes in enterohemorrhagic Escherichia coli O157: H7. Infect Immun. 2002;70(7):3500–3509. doi: 10.1128/IAI.70.7.3500-3509.2002 12065489 PMC128104

[52] Morgan JK, Carroll RK, Harro CM, et al. Global regulator of virulence a (GrvA) coordinates expression of discrete pathogenic mechanisms in enterohemorrhagic Escherichia coli through interactions with GadW-GadE. J Bacteriol. 2016;198(3):394–409. doi: 10.1128/JB.00556-15 26527649 PMC4719443

[53] Morgan JK, Vendura KW, Stevens SM, et al. RcsB determines the locus of enterocyte effacement (LEE) expression and adherence phenotype of Escherichia coli O157: H7 spinach outbreak strain TW14359 and coordinates bicarbonate-dependent LEE activation with repression of motility. Microbiol (Read). 2013;159(Pt 11):2342–2353. doi: 10.1099/mic.0.070201-0 23985143

[54] Beyhan S, Tischler AD, Camilli A, et al. Differences in gene expression between the classical and El tor biotypes of Vibrio cholerae O1. Infect Immun. 2006;74(6):3633–3642. doi: 10.1128/IAI.01750-05 16714595 PMC1479229

[55] Iwanaga M, Yamamoto K. New medium for the production of cholera toxin by vibrio cholerae O1 biotype El Tor. J Clin Microbiol. 1985;22(3):405–408. doi: 10.1128/jcm.22.3.405-408.1985 2995438 PMC268420

[56] Kotiranta A, Lounatmaa K, Haapasalo M. Epidemiology and pathogenesis of Bacillus cereus infections. Microbes Infect. 2000;2(2):189–198. doi: 10.1016/S1286-4579(00)00269-0 10742691

[57] Shimoyama Y, Umegaki O, Ooi Y, et al. Bacillus cereus pneumonia in an immunocompetent patient: a case report. JA Clin Rep. 2017;3(1):25. doi: 10.1186/s40981-017-0096-3 29457069 PMC5804607

[58] Helgason E, Økstad OA, Caugant DA, et al. Bacillus anthracis , Bacillus cereus , and Bacillus thuringiensis —one species on the basis of genetic evidence. Appl Environ Microbiol. 2000;66(6):2627–2630. doi: 10.1128/AEM.66.6.2627-2630.2000 10831447 PMC110590

[59] Sirard JC, Mock M, Fouet A. The three bacillus anthracis toxin genes are coordinately regulated by bicarbonate and temperature. J Bacteriol. 1994;176(16):5188–5192. doi: 10.1128/jb.176.16.5188-5192.1994 8051039 PMC196368

[60] Casey JR. Why bicarbonate?This paper is one of a selection of papers published in this special issue, entitled CSBMCB — membrane proteins in health and disease. Biochem Cell Biol. 2006;84(6):930–939. doi: 10.1139/o06-184 17215880

[61] Dai Z, Koehler TM. Regulation of anthrax toxin activator gene (atxA) expression in Bacillus anthracis: temperature, not CO2/bicarbonate, affects AtxA synthesis. Infect Immun. 1997;65(7):2576–2582. doi: 10.1128/iai.65.7.2576-2582.1997 9199422 PMC175364

[62] Jang J, Cho M, Chun J-H, et al. The poly-γ-D-glutamic acid capsule of bacillus anthracis enhances lethal toxin activity. Infect Immun. 2011;79(9):3846–3854. doi: 10.1128/IAI.01145-10 21690241 PMC3165481

[63] Drysdale M, Bourgogne A, Koehler TM. Transcriptional analysis of the bacillus anthracis capsule regulators. J Bacteriol. 2005;187(15):5108–5114. doi: 10.1128/JB.187.15.5108-5114.2005 16030203 PMC1196023

[64] Uchida I, Makino S-I, Sekizaki T, et al. Cross-talk to the genes for bacillus anthracis capsule synthesis by atxA, the gene encoding the trans-activator of anthrax toxin synthesis. Mol Microbiol. 1997;23(6):1229–1240. doi: 10.1046/j.1365-2958.1997.3041667.x 9106214

[65] Koehler TM, Dai Z, Kaufman-Yarbray M. Regulation of the bacillus anthracis protective antigen gene: CO2 and a trans-acting element activate transcription from one of two promoters. J Bacteriol. 1994;176(3):586–595. doi: 10.1128/jb.176.3.586-595.1994 8300513 PMC205094

[66] Passalacqua KD, Varadarajan A, Byrd B, et al. Comparative transcriptional profiling of Bacillus cereus sensu lato strains during growth in CO2-bicarbonate and aerobic atmospheres. PLoS One. 2009;4(3):e4904. doi: 10.1371/journal.pone.0004904 19295911 PMC2654142

[67] Scarff JM, Raynor MJ, Seldina YI, et al. The roles of AtxA orthologs in virulence of anthrax-like Bacillus cereus G9241. Mol Microbiol. 2016;102(4):545–561. doi: 10.1111/mmi.13478 27490458 PMC5118089

[68] Collins JW, Keeney KM, Crepin VF, et al. Citrobacter rodentium: infection, inflammation and the microbiota. Nat Rev Microbiol. 2014;12(9):612–623. doi: 10.1038/nrmicro3315 25088150

[69] Bourgogne A, Thomson LC, Murray BE. Bicarbonate enhances expression of the endocarditis and biofilm associated pilus locus, ebpR-ebpABC, in Enterococcus faecalis. BMC Microbiol. 2010;10(1):17. doi: 10.1186/1471-2180-10-17 20092636 PMC2824692

[70] Bourgogne A, Singh KV, Fox KA, et al. EbpR is important for biofilm formation by activating expression of the endocarditis and biofilm-associated pilus operon (ebpABC) of Enterococcus faecalis OG1RF. J Bacteriol. 2007;189(17):6490–6493. doi: 10.1128/JB.00594-07 17586623 PMC1951926

[71] Koehler TM. Bacillus anthracis genetics and virulence gene regulation. Curr Top Microbiol Immunol. 2002;271:143–164. 12224521 10.1007/978-3-662-05767-4_7

[72] Mogensen EG, Janbon G, Chaloupka J, et al. Cryptococcus neoformans senses CO2 through the carbonic anhydrase Can2 and the adenylyl cyclase Cac1. Eukaryot Cell. 2006;5(1):103–111. doi: 10.1128/EC.5.1.103-111.2006 16400172 PMC1360268

[73] Rose SJ, Bermudez LE, Kaufmann SHE. *I*dentification of bicarbonate as a trigger and genes involved with extracellular DNA export in mycobacterial biofilms. MBio. 2016;7(6). doi: 10.1128/mBio.01597-16 PMC5142616 27923918

[74] Johnson BK, Colvin CJ, Needle DB, et al. The carbonic anhydrase inhibitor ethoxzolamide inhibits the mycobacterium tuberculosis PhoPR regulon and esx-1 secretion and attenuates virulence. Antimicrob Agents Chemother. 2015;59(8):4436–4445. doi: 10.1128/AAC.00719-15 25987613 PMC4505220

[75] Aspatwar A, Hammarén M, Koskinen S, et al. β-ca-specific inhibitor dithiocarbamate Fc14–584B: a novel antimycobacterial agent with potential to treat drug-resistant tuberculosis. J Enzyme Inhib Med Chem. 2017;32(1):832–840. doi: 10.1080/14756366.2017.1332056 28629306 PMC6445161

[76] Siew R, Ou T-L, Dahesh S, et al. Bicarbonate effects on antibacterial immunity and mucus glycobiology in the cystic fibrosis lung: a review with selected experimental observations. Infect Microb Dis. 2022;4(3):103–110. doi: 10.1097/IM9.0000000000000101 PMC9928163 36793929

[77] Abbas G, Ahmad F, Saeed M, et al. Effect of dietary inclusion of sodium bicarbonate on digestibility of nutrients and immune response in caged layers during the summer. B J Poul Sci. 2019;21(2):21(2. doi: 10.1590/1806-9061-2018-0915

[78] Hewawaduge C, Senevirathne A, Lee JH. Enhancement of host infectivity, immunity, and protective efficacy by addition of sodium bicarbonate antacid to oral vaccine formulation of live attenuated salmonella secreting Brucella antigens. Microb Pathog. 2020;138:103857. doi: 10.1016/j.micpath.2019.103857 31705999

[79] Strowitzki MJ, Nelson R, Garcia MP, et al. Carbon dioxide sensing by immune cells occurs through carbonic anhydrase 2–dependent changes in intracellular pH. J Immunol. 2022;208(10):2363–2375. doi: 10.4049/jimmunol.2100665 35477686

[80] Helenius IT, Krupinski T, Turnbull DW, et al. Elevated CO2 suppresses specific drosophila innate immune responses and resistance to bacterial infection. Proc Natl Acad Sci U S A. 2009;106(44):18710–18715. doi: 10.1073/pnas.0905925106 19846771 PMC2773965

[81] Schneberger D, Pandher U, Thompson B, et al. Effects of elevated CO2 levels on lung immune response to organic dust and lipopolysaccharide. Respir Res. 2021;22(1):104. doi: 10.1186/s12931-021-01700-4 33836776 PMC8033726

[82] Dechow S, Goyal R, Johnson BJ, et al. Carbon dioxide regulates mycobacterium tuberculosis PhoPR signaling and virulence. bioRxiv. 2022. 10.1128/iai.00568-24 PMC11895460 39964175

[83] An Y, Ni R, Zhuang L, et al. Tuberculosis vaccines and therapeutic drug: challenges and future directions. Mol Biomed. 2025;6(1):4. doi: 10.1186/s43556-024-00243-6 39841361 PMC11754781

[84] Parkkinen J, Bhowmik, R., Tolvanen, M., Carta, F., Supuran, C.T., Parkkila, S. and Aspatwar, A. Mycobacterial β-carbonic anhydrases: molecular biology, role in the pathogenesis of tuberculosis and inhibition studies. Enzymes. 2024;55:343–381. 39222997 10.1016/bs.enz.2024.05.012

[85] Supuran CT. Bacterial carbonic anhydrases as drug targets: toward novel antibiotics? Front Pharmacol. 2011;2:34. doi: 10.3389/fphar.2011.00034 21779249 PMC3132667

[86] Chilambi GS, Wang Y-H, Wallace NR, et al. Carbonic anhydrase inhibition as a target for antibiotic synergy in enterococci. Microbiol Spectr. 2023;11(4):e0396322. doi: 10.1128/spectrum.03963-22 37260400 PMC10434275

[87] Supuran CT. Novel carbonic anhydrase inhibitors for the treatment of Helicobacter pylori infection. Expert Opin Investig Drugs. 2024;33(5):523–532. doi: 10.1080/13543784.2024.2334714 38517734

[88] Stähler FN, Ganter L, Lederer K, et al. Mutational analysis of the Helicobacter pylori carbonic anhydrases. FEMS Immunol Med Microbiol. 2005;44(2):183–189. doi: 10.1016/j.femsim.2004.10.021 15866214

[89] Supuran CT. Carbonic anhydrases: novel therapeutic applications for inhibitors and activators. Nat Rev Drug Discov. 2008;7(2):168–181. doi: 10.1038/nrd2467 18167490

[90] Nocentini A, Capasso C, Supuran CT. Carbonic anhydrase inhibitors as novel Antibacterials in the era of antibiotic resistance: where are we Now? Antibiotics (Basel). 2023;12(1):12(1. doi: 10.3390/antibiotics12010142 PMC9854953 36671343

[91] Aspatwar A, Hammaren M, Parikka M, et al. In vitro inhibition of mycobacterium tuberculosis β -carbonic anhydrase 3 with mono- and dithiocarbamates and evaluation of their toxicity using zebrafish developing embryos. J Enzyme Inhib Med Chem. 2020;35(1):65–71. doi: 10.1080/14756366.2019.1683007 31663386 PMC6830242

[92] Bhowmik R, Vyas A, Manaithiya B, et al. Navigating bioactivity space in anti-tubercular drug discovery through the deployment of advanced machine learning models and cheminformatics tools: a molecular modeling based retrospective study. Front Pharmacol. 2023;14:1265573. doi: 10.3389/fphar.2023.1265573 37705534 PMC10495588

